# Factors associated with return to pre-admission functional category at hospital discharge among acute inpatients receiving physical therapy and/or occupational therapy: A retrospective cohort study

**DOI:** 10.1371/journal.pone.0355836

**Published:** 2026-08-14

**Authors:** Yazeed Temraz, Wafa Bin Salamh, Naif Alsayahi, Mohammed Alrehaili, Fahdah Aljamaan, Hend Alqudaimi, Dina Alqueflie

**Affiliations:** 1 Rehabilitation Department, Ministry of National Guard Health Affairs, Riyadh, Saudi Arabia; 2 King Abdullah International Medical Research Center, Riyadh, Saudi Arabia; Mayo Clinic College of Medicine and Science, UNITED STATES OF AMERICA

## Abstract

**Background:**

Functional decline associated with acute hospitalization may persist at hospital discharge. This study examined associations between routinely documented admission-time characteristics and return to the same pre-admission functional category among acute inpatients receiving physical therapy and/or occupational therapy.

**Methods:**

This exploratory single-center retrospective cohort study included 444 unique adults who received acute inpatient physical therapy and/or occupational therapy at a tertiary hospital. Ninety patients classified as bedridden before admission were excluded because no outcome variation was observed in that stratum. Modified Poisson regression with robust variance was used to estimate adjusted risk ratios (aRRs) for return to the same pre-admission documentation-derived functional category at discharge.

**Results:**

The primary cohort included 354 patients, of whom 146 (41.2%) returned to the same pre-admission documentation-derived functional category at discharge. Each one-level worsening in admission functional severity was associated with a lower probability of return (aRR 0.59, 95% confidence interval [CI] 0.47–0.75). In the exploratory subgroup of 164 patients independently ambulatory before admission, worse admission functional severity was associated with a higher risk of discharge below the pre-admission category (aRR 1.17, 95% CI 1.03–1.32). Associations with premorbid functional category reflected different premorbid functional targets and were not interpreted as comparative recovery probabilities.

**Conclusions:**

Routinely documented premorbid and admission functional information may help identify acute inpatients receiving physical therapy and/or occupational therapy who may be at risk of leaving hospital below their pre-admission functional category. Because the endpoint represents no net decline relative to premorbid function and reflects different targets across baseline strata, findings should not be interpreted as evidence that patients with greater premorbid dependency recover better.

## 1. Introduction

Functional decline during and after acute hospitalization is common and clinically important, particularly among older adults and patients with complex medical conditions [1–4]. A meta-analysis of 15 studies estimated that approximately 30% of hospitalized older adults experience hospital-associated disability, defined as loss of independence in activities of daily living following acute hospitalization [5]. Patients discharged below their pre-admission functional level may require greater support after discharge and may experience adverse longer-term outcomes, including institutionalization and mortality [1–4,6].

Hospital-associated deconditioning is not uniformly defined across the literature and may involve mobility, self-care, cognition, and other domains [4,7]. Previous studies have examined functional decline and recovery across older-adult acute-care, cardiac, intensive-care, post-acute, and inpatient-rehabilitation populations; however, their settings, functional measures, and follow-up periods vary substantially. In a prospective cohort of older adults admitted to a cardiac care unit, admission functional assessment was used to predict hospitalization-associated functional decline [8]. In a population-level cohort of more than 80,000 hospitalized older adults, discharge functional status improved prediction of long-term care placement and mortality [9]. In inpatient rehabilitation populations, functional status outperformed demographic and comorbidity-based models for predicting 30-day acute-care readmission [10], while a large national stroke-rehabilitation dataset identified admission Functional Independence Measure motor score as a major predictor of discharge outcomes [11].

Premorbid function is also clinically relevant. A systematic review and meta-analysis identified cognitive impairment and delirium as statistically supported patient-related risk factors for in-hospital functional decline among older adults; its narrative synthesis also identified prehospital functional decline and need for walking assistance [12]. Among very old intensive-care patients, poor premorbid functional status has been associated with mortality and subsequent functional outcomes [13]. However, the association between routinely documented premorbid and admission functional status and return to the same premorbid category at acute-hospital discharge has been less clearly described among mixed adult inpatients receiving physical therapy and/or occupational therapy.

Return to the same pre-admission functional category is not a uniform recovery target. A patient who was independently ambulatory before admission must regain independent ambulation to meet that endpoint, whereas a patient who was chairbound before admission may meet the endpoint by returning to chair-level mobility. This structural asymmetry requires explicit consideration when interpreting associations across premorbid functional strata.

This exploratory, single-center retrospective cohort study aimed to estimate associations between routinely documented admission-time demographic, clinical, and functional characteristics and return to the same pre-admission documentation-derived functional category at hospital discharge among adult acute inpatients receiving physical therapy and/or occupational therapy in a tertiary-hospital setting.

## 2. Materials and methods

### 2.1. Study design, setting, and data source

This exploratory, single-center retrospective cohort study used routinely collected electronic health record (EHR) data from adult acute inpatients at a tertiary hospital. Reporting was guided by the Strengthening the Reporting of Observational Studies in Epidemiology (STROBE) statement for cohort studies [14].

Source records included admission notes, nursing documentation, physical therapy (PT) and occupational therapy (OT) notes recorded during the hospital stay, discharge documentation, and structured EHR-coded demographic, diagnostic, comorbidity, and service-use data. Structured variables were extracted from coded EHR fields by the study biostatistician. Documentation-derived clinical information, including functional status, was reviewed, cleaned, and coded by six study assessors using predefined variable definitions.

The source cohort included patients admitted from 1 January 2023–31 December 2024, with follow-up through hospital discharge. The latest discharge date was 7 March 2025.

### 2.2. Participants and cohort construction

The source cohort comprised 444 unique adult patients aged 18 years or older who received PT and/or OT during an acute inpatient admission. Eligibility required at least one documented PT and/or OT assessment or intervention during the index admission. Each record represented one patient and one index admission; repeated admissions were not present in the analytical dataset.

Patients classified as bedridden before admission were excluded from the primary analysis because all were also classified as bedridden at discharge, resulting in no observed outcome variation within that stratum. After exclusion of these 90 patients, the primary analytic cohort included 354 patients.

An exploratory secondary subgroup analysis was restricted to patients who had been independently ambulatory before admission (n = 164).

### 2.3. Documentation-derived functional status, outcome, and predictors

Functional status was abstracted chronologically across the index hospital admission. Pre-admission functional status was obtained from functional history recorded in the admission note. Admission functional status was identified from the earliest nursing intake documentation and the first available PT and OT assessments after admission. Subsequent nursing and rehabilitation documentation was reviewed to confirm the recorded functional trajectory. Discharge functional status was obtained from final discharge documentation, with final nursing or rehabilitation documentation used as supporting evidence where available.

Functional categories were derived from routine clinical documentation by six assessors using predefined operational definitions presented in Supplementary Table S1 in S1 File. The abstraction process was intended to reconstruct the chronological documentation record rather than provide an independent blinded functional assessment. Formal blinding to discharge status, duplicate independent abstraction, and inter-rater reliability testing were not undertaken. No conflicting functional documentation requiring adjudication was identified.

The primary outcome was return to the same pre-admission documentation-derived functional category at hospital discharge, defined as agreement between pre-admission and discharge functional categories. Functional status was classified as ambulatory, ambulatory with assistance, chairbound, bedbound, or bedridden. Ambulatory indicated independent mobility on level surfaces, with or without a usual mobility aid and without physical assistance. Ambulatory with assistance indicated a requirement for supervision, contact guard, physical assistance, or another person for safe walking. Chairbound indicated inability to ambulate functionally despite the ability to sit out of bed. Bedbound indicated confinement mainly to bed with possible supported sitting or limited transfer. Bedridden indicated inability to mobilize out of bed or transfer meaningfully.

For the primary analysis, chairbound and bedbound categories were combined because both represented severe non-ambulatory dependence and separate categories had limited cell sizes. The bedridden baseline stratum was excluded from the primary analysis because no observed outcome variation was present within that group.

Candidate admission-time predictors were age, sex, body mass index, diagnosis group, comorbidity count, documented cognitive impairment at admission, premorbid documentation-derived functional stratum, and admission functional severity. Documented cognitive impairment was a binary documentation-based proxy abstracted from the admission note. It was coded as present when cognitive impairment was documented by the admitting clinical team and absent when no such documentation was recorded. Because this measure was not based on a standardized cognitive assessment, it was interpreted as a pragmatic documentation-based proxy that may have been under-recorded or misclassified.

Diagnosis groups were cardiovascular, infection, musculoskeletal or trauma, neurologic, and other. Neurologic and other diagnoses were combined for regression analyses because of sparse cell counts. Functional Independence Measure (FIM) and Barthel Index scores were directly recorded standardized measures in PT and OT documentation as part of routine rehabilitation practice; they were not reconstructed, imputed, or mapped from the five-category functional classification.

Because return to the same pre-admission category represents different functional targets across premorbid strata, the outcome was interpreted as no net decline relative to premorbid function rather than equivalent clinical recovery across all groups. The exploratory ambulatory subgroup was examined because these patients shared a common premorbid target of independent ambulation. Its outcome was discharge below the pre-admission documentation-derived functional category.

Post-admission variables, including in-hospital complications, early mobilization within 48 hours, length of stay, and therapy-session counts, were summarized descriptively in Supplementary Table S3 in S1 File and were not included in the admission-time association models.

### 2.4. Data quality, missing data, and measurement limitations

A structured data-quality review was undertaken before analysis. Internal consistency checks confirmed agreement between admission and discharge dates and recorded length of stay, between PT plus OT sessions and total therapy sessions, and between recorded pre-admission and discharge functional categories and the derived return-to-pre-admission-category outcome.

All variables included in the primary multivariable model were complete; therefore, no imputation was performed. Four records had missing free-text comorbidity-list information, although coded comorbidity count was complete for all records and was used in the primary model. Complication type was not applicable for patients without a documented complication; no true missing values were present for this variable. The exploratory ambulatory-subgroup outcome was structurally undefined for patients who had not been independently ambulatory before admission. Missing-data and quality-control results are reported in Supplementary Table S2 in S1 File.

The use of documentation-derived functional categories may have introduced measurement error. Although predefined coding rules were used, formal inter-rater reliability testing was not undertaken.

### 2.5. Statistical analysis

Continuous variables were summarized as median and interquartile range, and categorical variables as number and percentage.

The primary association analysis used modified Poisson regression with robust variance estimation to estimate adjusted risk ratios (aRRs) and 95% confidence intervals for return to the same pre-admission documentation-derived functional category at discharge. This approach was selected because the primary outcome was common and logistic-regression odds ratios could overstate the magnitude of association [15–18].

The primary multivariable model included sex, diagnosis group, premorbid documentation-derived functional stratum, age per 10-year increment, body mass index per 5 kg/m² increment, comorbidity count, documented cognitive impairment at admission, and admission functional severity. The primary model included 354 eligible patients and 146 return-to-pre-admission-category events. Neurologic and other diagnoses were combined because of sparse cell counts.

The exploratory ambulatory subgroup analysis used modified Poisson regression with robust variance to estimate risk ratios for discharge below pre-admission functional status among patients independently ambulatory before admission. This subgroup included 164 patients, of whom 118 experienced discharge decline.

Sensitivity analyses replaced ordinal admission functional severity with either admission FIM score or Barthel Index score. These measures were treated as alternative parameterizations of admission severity because of their high correlation and were not entered in the same model as ordinal admission severity. A logistic regression model using the same primary predictor set was fitted as a model-form sensitivity analysis. Odds ratios from this model were interpreted only for consistency of direction and inference and were not compared directly with primary risk ratios. An additional exploratory sensitivity model separated chairbound and bedbound baseline categories to assess potential heterogeneity within the pooled severe-dependence stratum.

Primary-model diagnostics assessed convergence, fitted-value range, variance inflation factors, influence using Cook’s distance, and potential non-linearity using quadratic terms for age, body mass index, and comorbidity count. A sensitivity analysis excluded observations with Cook’s distance greater than 4/n. Diagnostic and robustness results are reported in Supplementary Table S8 in S1 File.

Two-sided p values below 0.05 were considered nominally statistically significant. Given the exploratory nature of the analyses, no multiplicity adjustment was applied; interpretation prioritised effect estimates and 95% confidence intervals. No formal a priori or post hoc sample-size calculation was performed because the full available retrospective cohort was used. Precision was assessed using 95% confidence intervals, with particular caution applied to sparse diagnosis categories and exploratory subgroup estimates.

Complete regression coefficients, robust standard errors, confidence intervals, exact p values, and model-fit information are provided in the supplementary materials. Statistical analyses were performed in Python 3.13.5 using pandas 2.2.3, NumPy 2.3.5, and statsmodels 0.14.6.

### 2.6. Ethics statement

This study was reviewed and approved by the institutional review board (IRB; approval no. 00000178925). The requirement for informed consent was waived because the study was retrospective and based on routinely collected EHR data. Data were extracted in September 2025 and handled in de-identified format before analysis. All procedures were conducted in accordance with applicable institutional policies and relevant ethical standards for human research, including the Declaration of Helsinki.

## 3. Results

### 3.1. Cohort flow and baseline characteristics

**Fig 1:**
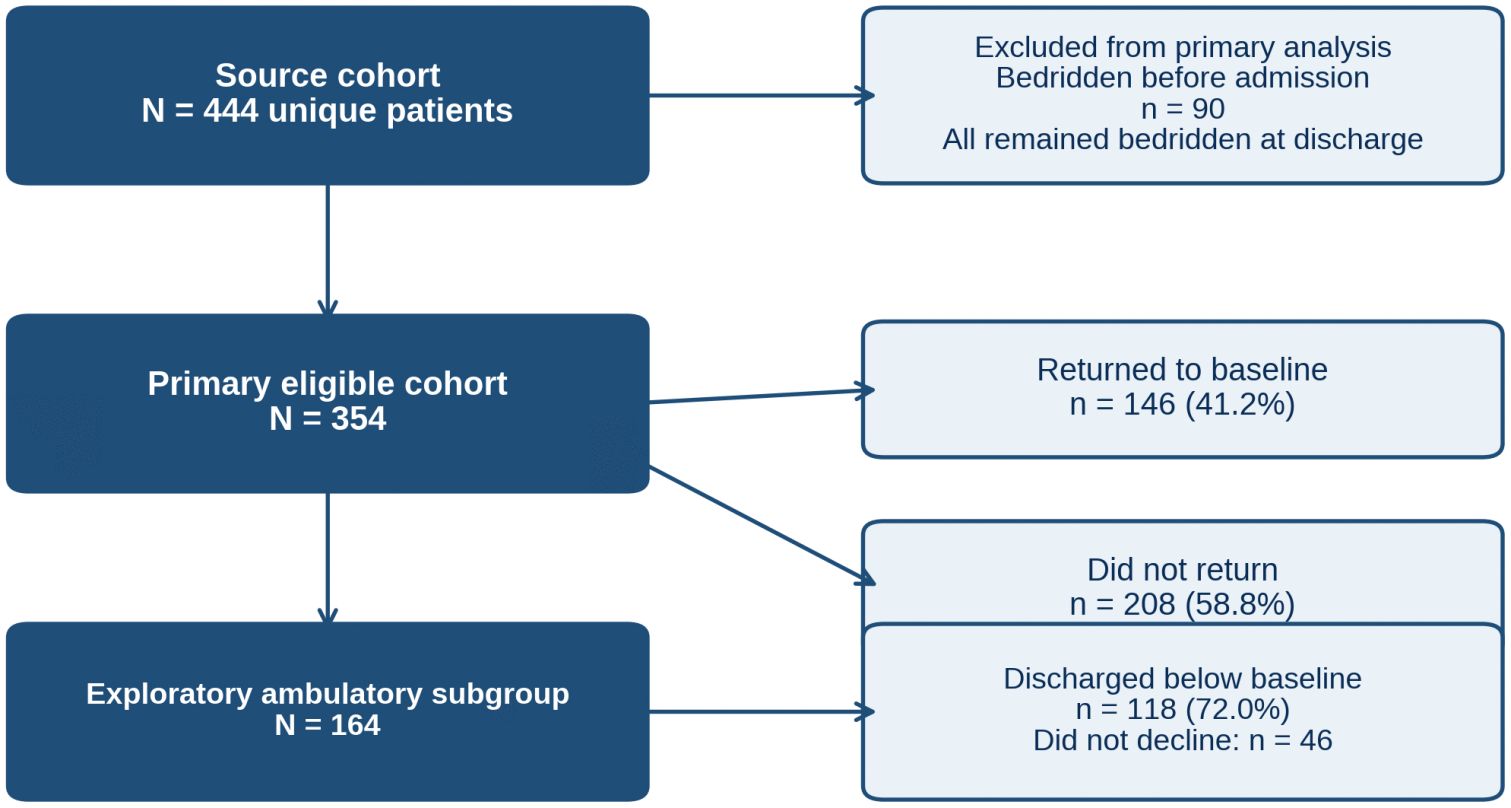
Participant flow through the primary and exploratory secondary analytic cohorts. Patients classified as bedridden before admission were also classified as bedridden at discharge, resulting in no observed outcome variation within that stratum.

The source cohort comprised 444 unique adult patients. Ninety patients classified as bedridden before admission were excluded from the primary analysis because all were also classified as bedridden at discharge, resulting in no observed outcome variation within that stratum. The primary cohort therefore included 354 patients, of whom 146 (41.2%) returned to the same pre-admission documentation-derived functional category at discharge and 208 (58.8%) did not. Participant flow is shown in Fig 1.

The exploratory subgroup of patients who had been independently ambulatory before admission included 164 patients; 118 (72.0%) were discharged below their pre-admission documentation-derived functional category.

In the primary cohort, median age was 68.0 years [interquartile range, 55.2–78.0], and 184 patients (52.0%) were female. Cardiovascular, infection, and musculoskeletal or trauma diagnoses each accounted for approximately one-third of the cohort. Return to the recorded pre-admission category was observed in 28.0% of patients who had been independently ambulatory before admission, 45.9% of those ambulatory with assistance, and 59.8% of those chairbound or bedbound. Because patients began with different premorbid functional targets, these proportions should not be interpreted as comparative recovery rates (Table 1).

**Table 1:** Baseline characteristics of the primary cohort according to return to the same pre-admission functional category at hospital discharge.

Variable	Overall	Returned to the same pre-admissionfunctional category	Did not return to the pre-admissionfunctional category
Age, years	68.0 [55.2, 78.0]	71.0 [59.0, 81.0]	66.0 [54.0, 76.0]
BMI, kg/m²	29.3 [26.9, 32.3]	29.4 [26.9, 32.3]	29.3 [26.9, 32.0]
Comorbidity count	5.0 [4.0, 7.8]	6.0 [4.0, 8.0]	5.0 [3.0, 7.0]
FIM admission score	93.0 [40.0, 110.0]	78.0 [45.0, 110.0]	94.5 [32.0, 109.2]
Barthel admission score	75.0 [30.0, 85.0]	65.0 [35.0, 85.0]	77.5 [15.0, 85.0]
Admission function ordinal score	2.0 [2.0, 4.0]	3.0 [2.0, 4.0]	2.0 [2.0, 5.0]
**Sex**			
*- Female*	184 (52.0%)	88 (60.3%)	96 (46.2%)
*- Male*	170 (48.0%)	58 (39.7%)	112 (53.8%)
**Diagnosis group (descriptive, 5 categories)**			
*- Cardiovascular*	114 (32.2%)	44 (30.1%)	70 (33.7%)
*- Infection*	113 (31.9%)	46 (31.5%)	67 (32.2%)
*- Musculoskeletal/Trauma*	112 (31.6%)	49 (33.6%)	63 (30.3%)
*- Neurologic*	9 (2.5%)	5 (3.4%)	4 (1.9%)
*- Other*	6 (1.7%)	2 (1.4%)	4 (1.9%)
Pre-admission documentation-derived functional stratum			
*- Ambulatory*	164 (46.3%)	46 (31.5%)	118 (56.7%)
*- Ambulatory with Assistance*	98 (27.7%)	45 (30.8%)	53 (25.5%)
*- Chairbound/Bedbound*	92 (26.0%)	55 (37.7%)	37 (17.8%)
Documented cognitive impairment at admission			
*- No*	262 (74.0%)	110 (75.3%)	152 (73.1%)
*- Yes*	92 (26.0%)	36 (24.7%)	56 (26.9%)

Data are presented as n (%) or median [interquartile range]. Pre-admission documentation-derived functional categories were abstracted from routine clinical records. Patients classified as bedridden before admission were excluded from the primary cohort because no outcome variation was observed in that stratum. Neurologic and other diagnoses were combined in regression analyses because of sparse cell counts. Differences in admission Functional Independence Measure and Barthel Index scores between outcome groups should be interpreted in the context of differing premorbid functional targets.

### 3.2. Primary association analysis

In the adjusted modified Poisson model, worse admission functional severity was associated with a lower probability of return to the same pre-admission documentation-derived functional category at discharge (adjusted risk ratio [aRR] 0.59 per one-level worsening, 95% confidence interval [CI] 0.47–0.75; Table 2).

**Table 2:** Crude and adjusted risk ratios for return to the same pre-admission functional category at hospital discharge in the primary cohort.

Predictor	Crude RR (95% CI)	Crude P	Adjusted RR (95% CI)	Adjusted P
Male sex (vs female)	0.71 (0.55-0.92)	0.010	0.82 (0.64-1.06)	0.125
Diagnosis: Infection (vs Cardiovascular)	1.05 (0.77-1.45)	0.745	1.36 (0.87-2.10)	0.174
Diagnosis: Musculoskeletal/Trauma (vs Cardiovascular)	1.13 (0.83-1.55)	0.432	1.25 (0.93-1.68)	0.135
Diagnosis: Neurologic/Other (vs Cardiovascular)	1.21 (0.67-2.18)	0.527	1.60 (0.97-2.65)	0.066
Pre-admission category: ambulatory with assistance (vs ambulatory)	1.64 (1.18-2.27)	0.003	2.34 (1.62-3.38)	<0.001
Pre-admission category: chairbound/bedbound (vs ambulatory)	2.13 (1.58-2.87)	<0.001	6.25 (3.56-10.97)	<0.001
Age, per 10 years	1.13 (1.04-1.23)	0.003	1.03 (0.93-1.14)	0.552
BMI, per 5 kg/m²	0.98 (0.82-1.16)	0.795	1.06 (0.91-1.24)	0.444
Comorbidity count, per 1	1.09 (1.04-1.13)	<0.001	1.01 (0.96-1.07)	0.606
Documented cognitive impairment at admission (yes vs no)	0.93 (0.70-1.25)	0.636	0.98 (0.73-1.31)	0.871
Admission function ordinal level, per 1-level worse	0.95 (0.86-1.04)	0.280	0.59 (0.47-0.75)	<0.001

Risk ratios and 95% confidence intervals were estimated using modified Poisson regression with robust variance. The primary analysis excluded patients classified as bedridden before admission. Associations for pre-admission functional category are structurally coupled to the outcome definition because patients began with different premorbid functional targets; these estimates should not be interpreted as comparative recovery probabilities.

Associations for premorbid functional strata are also reported in Table 2. However, these estimates are structurally coupled to the outcome definition because patients began with different premorbid functional targets. They should therefore not be interpreted as comparative recovery probabilities.

Estimates for diagnosis group were imprecise, particularly for the combined neurologic or other category. Age, sex, body mass index, comorbidity count, and documented cognitive impairment at admission did not show clear independent associations with the outcome after adjustment.

### 3.3. Exploratory ambulatory-baseline subgroup analysis

Among patients who had been independently ambulatory before admission, musculoskeletal or trauma diagnosis was associated with a lower risk of discharge decline than cardiovascular diagnosis (aRR 0.78, 95% CI 0.62–0.98). Worse admission functional severity was associated with a higher risk of discharge below the pre-admission documentation-derived functional category (aRR 1.17, 95% CI 1.03–1.32).

Because this was an exploratory subgroup analysis, these estimates should be interpreted cautiously. Descriptive post-admission hospital-course variables are reported in Supplementary Table S3 in S1 File.

### 3.4. Sensitivity analyses

When ordinal admission functional severity was replaced with directly documented admission Functional Independence Measure (FIM) or Barthel Index scores, findings were directionally consistent. Higher admission FIM score was associated with a higher probability of return to the same pre-admission documentation-derived functional category at discharge (aRR 1.15 per 10-point increase, 95% CI 1.06–1.24; p < 0.001). Similarly, higher admission Barthel Index score was associated with a higher probability of return to the pre-admission category (aRR 1.20 per 10-point increase, 95% CI 1.10–1.31; p < 0.001).

Because FIM, Barthel Index, and ordinal admission severity arose from the same clinical rehabilitation documentation stream and were highly correlated, these analyses were interpreted as alternative parameterizations of admission functional severity rather than independent validation using distinct constructs (Supplementary Tables S4 and S5 in S1 File).

The logistic-regression sensitivity analysis showed the same overall direction of association for premorbid functional stratum and admission functional severity (Supplementary Table S6 in S1 File). An exploratory sensitivity analysis separating chairbound from bedbound baseline categories is reported in Supplementary Table S7 in S1 File. Complete regression output is also provided in Supplementary Table S7 in S1 File.

### 3.5. Model diagnostics and robustness checks

The primary modified Poisson model converged successfully. Diagnostic analyses showed no material collinearity or evidence of non-linearity for age, body mass index, or comorbidity count. Excluding influential observations did not alter the direction of the association between worse admission functional severity and return to the same pre-admission documentation-derived functional category. Complete diagnostic and robustness results are reported in Supplementary Table S8 in S1 File.

## 4. Discussion

This exploratory, single-center retrospective cohort study found that worse documentation-derived admission functional severity was associated with a lower probability of return to the same pre-admission functional category at hospital discharge among acute inpatients receiving physical therapy and/or occupational therapy. The most clinically interpretable finding was observed in the exploratory subgroup of patients who had been independently ambulatory before admission: worse admission functional severity was associated with a higher risk of discharge below independent ambulation. These findings do not establish causal effects of rehabilitation treatment and do not constitute development or validation of a prognostic prediction model.

The findings are consistent with previous evidence showing that functional status documented during hospitalization is associated with clinically important outcomes. In inpatient rehabilitation populations, functional status has been associated with 30-day readmission risk, and in stroke-rehabilitation cohorts, admission functional measures have been associated with discharge outcomes [10,11]. Premorbid functional impairment has also been identified as a risk factor for in-hospital functional decline in older adults, while poor premorbid function has been associated with mortality and functional outcomes among very old intensive-care patients [12,13]. The 41.2% rate of return to the pre-admission category in this cohort should not be compared directly with estimates from studies using different populations, functional measures, care settings, or follow-up periods. For example, previous studies have examined recovery among older adults admitted to nursing homes after acute hospitalization or among those receiving home health care after skilled nursing facility discharge, with follow-up over several months rather than functional status at acute-hospital discharge [2,19–21].

Return to the same pre-admission documentation-derived functional category represents no net decline relative to premorbid function; it does not indicate equivalent clinical recovery across patients with different premorbid dependency levels. Patients who had been independently ambulatory before admission needed to regain independent ambulation to meet the endpoint, whereas patients who were chairbound or bedbound before admission could meet the endpoint by returning to that lower functional level. All patients classified as bedridden before admission were also classified as bedridden at discharge, resulting in no observed outcome variation within that stratum. Accordingly, associations for premorbid functional strata in the all-cohort model were partly shaped by different functional targets and should not be interpreted as comparative recovery probabilities. The ambulatory-baseline subgroup reduced, but did not eliminate, this structural asymmetry because all patients shared a common premorbid target of independent ambulationatory-baseline subgroup reduced, but did not eliminate, this structural asymmetry because all patients shared a common premorbid target of independent ambulation.

FIM and Barthel Index scores were directly recorded standardized measures in physical therapy and occupational therapy documentation and were not reconstructed from the five-category functional classification. However, FIM, Barthel Index, and ordinal admission functional severity were derived from the same routine rehabilitation documentation stream and represented closely related aspects of admission function. Because FIM and Barthel Index assess overlapping activities of daily living and disability domains [22], their near-perfect correlations in this cohort support interpretation as alternative parameterizations of the same documentation-derived admission-severity construct rather than independent validation using distinct constructs.

Age, sex, body mass index, comorbidity count, and documented cognitive impairment did not show clear independent associations after adjustment for functional status. This should not be interpreted as evidence that these factors lack clinical importance. It is plausible that admission functional status captured some aspects of illness burden and physiological reserve, although this could not be directly assessed in the present dataset. Other cohorts have reported associations of frailty, dynamic in-hospital factors, and multidimensional admission assessments with functional trajectories or rehabilitation outcomes [12,13,23,24].

This study has several strengths. It included the full available cohort of acute inpatients receiving rehabilitation services, used routinely documented functional information from multiple clinical records, and applied transparent cohort-construction and outcome definitions. The use of modified Poisson regression allowed direct estimation of risk ratios for a common outcome. Model diagnostics and influence sensitivity analyses were also undertaken and reported.

Several limitations should be considered. First, the retrospective single-center design limits causal interpretation and transferability. The cohort was drawn from a tertiary hospital in Saudi Arabia and included acute inpatients referred for and receiving physical therapy and/or occupational therapy. Referral thresholds, rehabilitation staffing, discharge-support systems, family-care structures, and access to post-acute rehabilitation may differ across healthcare settings.

Second, functional categories were derived from routine documentation and were less granular than standardized performance-based assessments. Although predefined coding rules were used, duplicate independent abstraction, formal assessor calibration, blinded coding, and inter-rater reliability assessment were not undertaken. Documented cognitive impairment was based on admission-note documentation rather than standardized cognitive testing and may have been under-recorded or misclassified.

Third, detailed indicators of acute illness severity, frailty, physiological instability, and some rehabilitation-dose characteristics were unavailable. Sparse counts in neurologic and other diagnosis categories limited precision. In addition, no patient improved beyond the recorded pre-admission functional category, meaning that the study evaluated return to premorbid status rather than functional gain beyond baseline.

In clinical practice, routinely documented premorbid and admission functional information may help prompt earlier multidisciplinary discharge discussions for patients at risk of leaving hospital below their prior functional category. However, implementation would require consistent functional definitions, reliable multidisciplinary documentation, and external validation before these findings are incorporated into formal prognostic tools, staffing decisions, or service-performance indicators.

## 5. Conclusions

Among acute adult inpatients receiving physical therapy and/or occupational therapy, worse documentation-derived admission functional severity was associated with a lower probability of return to the same pre-admission functional category at hospital discharge. Routine premorbid and admission functional information may help identify patients at risk of leaving hospital below their prior category. Associations for premorbid functional strata should not be interpreted as comparative recovery probabilities because the endpoint reflected different premorbid functional targets across dependency levels.

### AI use transparency

Generative AI tools were used for language editing and to assist in the drafting of the manuscript. The authors take full responsibility for the accuracy and originality of the content. AI tools are not listed as authors.

## Supporting information

S1 File Supplementary tables. Operational definitions, missing-data and data-quality summary, descriptive hospital-course findings, sensitivity analyses, correlation analysis, full regression output, and primary-model diagnostics.
(DOCX)

S2 Data Public anonymized individual-level dataset and variable codebook. Microsoft Excel workbook containing README, Data, and Codebook sheets.
(XLSX)

S3 Data Public anonymized individual-level dataset. Comma-separated values file used by the public analysis code.
(CSV)

S4 Code Public analysis code. Python script for public privacy-minimized analyses and, when provided with an approved restricted dataset, the fully adjusted models and primary-model diagnostics.
(PY)

S5 File Code README. Instructions for reproducing analyses and descriptions of public and controlled-access data, including model diagnostics.
(TXT)
