# Supplementary material for: Factors associated with return to pre-admission functional category at hospital discharge among acute inpatients receiving physical therapy and/or occupational therapy: A retrospective cohort study
Source: PLoS One. 2026 Aug 14;21(8):e0355836. doi: 10.1371/journal.pone.0355836 (PMC13475899; doi:10.1371/journal.pone.0355836)
Supplement: Supplementary file 1 [file pone.0355836.s001.docx]

**Supplementary Tables S1-S8**

**Table S1. Data sources and operational definitions of key study variables**

| **Variable** | **Source record(s)** | **Operational definition / handling** |
| --- | --- | --- |
| Pre-admission functional status | Admission note | Functional history recorded by the admitting team; classified as ambulatory, ambulatory with assistance, chairbound, bedbound, or bedridden. |
| Admission functional status | Nursing intake record; available PT and OT notes | Current functional status at hospital admission; classified using the same five functional categories. |
| Discharge functional status | Discharge note; supporting nursing or rehabilitation documentation where available | Final functional status at discharge; classified using the same five functional categories. |
| Return to pre-admission category | Derived from baseline and discharge functional categories | Agreement between pre-admission and discharge categories. |
| Documented cognitive impairment at admission | Admission note | Binary documentation-based proxy; coded present when cognitive impairment was documented by the admitting clinical team and absent when not documented. |
| Diagnosis group | Structured EHR-coded data extracted by the biostatistician | Cardiovascular, infection, musculoskeletal/trauma, neurologic, or other; neurologic and other were combined in regression analyses because of sparse counts. |
| Comorbidity count | Structured EHR-coded data extracted by the biostatistician | Count of coded comorbidities; used rather than the free-text comorbidity list in the primary model. |
| FIM and Barthel Index scores | Physical therapy and occupational therapy notes in the EHR | Directly recorded standardized admission scores; not reconstructed, imputed, or mapped from the five-category functional classification. Used only as alternative parameterizations of admission functional severity in sensitivity analyses. |
| Any improvement from admission to discharge | Derived from admission and discharge documentation-derived functional categories | Discharge functional category better than the admission category on the five-level ordinal scale (numerically lower ordinal score). |
| Hospital-course variables (overview) | Nursing and rehabilitation documentation; structured EHR fields | Length of stay, therapy sessions, early mobilization, and in-hospital complications. These variables occurred after admission and were described only; they were not included in the primary admission-time association model. |

**Table S1. Continued.**

| **Variable** | **Source record(s)** | **Operational definition / handling** |
| --- | --- | --- |
| Early mobilization within 48 h | Daily nursing records; first available PT or OT documentation | First documented out-of-bed activity within 48 hours of hospital admission. Out-of-bed activity included transfer from bed to chair, standing, walking, or another documented activity requiring mobilisation out of bed. The 48-hour threshold was used as a descriptive early-hospital-course timing marker and was not treated as an admission-time predictor or causal exposure. |
| In-hospital complication | Routine EHR documentation: coded diagnoses, progress notes, investigation results, and discharge summaries | Occurrence of one or more clinically documented adverse events during the index hospital admission. The composite included pneumonia, urinary tract infection, venous thromboembolism (deep-vein thrombosis or pulmonary embolism), cardiac events, and inpatient falls. Patients with at least one qualifying documented event were classified as having an in-hospital complication; patients with no documented qualifying event were classified as having no in-hospital complication. |

Functional category definitions: ambulatory = independently mobile on level surfaces, with or without a usual mobility aid and without physical assistance; ambulatory with assistance = requires supervision, contact guard, physical assistance, or another person for safe walking; chairbound = unable to ambulate functionally but able to sit out of bed; bedbound = mainly confined to bed but may tolerate supported sitting or limited transfer; bedridden = remains in bed and cannot mobilize out of bed or transfer meaningfully. Functional categories were documentation-derived from chronologically reviewed records; no source-prioritisation hierarchy was required because no conflicting functional documentation requiring adjudication was identified.

**Table S2. Missing-data and data-quality summary**

| **Item** | **Result** | **Interpretation** |
| --- | --- | --- |
| Source cohort | 444 records | All records included in the data-quality review. |
| Primary-model variables | 0 missing values | No imputation was performed. |
| Free-text comorbidity list | 4 missing records (0.9%) | Not used in the primary model; coded comorbidity count was complete. |
| Complication type | 389 not applicable; 0 true missing values | Not applicable when no in-hospital complication was documented; complication type was complete for all records with a documented complication. |
| Ambulatory-subgroup secondary outcome | 280 structurally undefined records (63.1%) | Defined only for patients independently ambulatory before admission. |
| Admission/discharge dates versus length of stay | Consistent in 444 records | Internal consistency check passed. |
| PT + OT sessions versus total therapy sessions | Consistent in 444 records | Internal consistency check passed. |
| Return-to-pre-admission-category outcome logic | Consistent in 444 records | Derived outcome matched equality of baseline and discharge categories. |
| Improvement beyond pre-admission category | 0 records | The outcome represented return to the same pre-admission functional category versus persistent decline. |

**Table S3. Descriptive hospital-course variables by return-to-pre-admission-functional-category outcome**

| **Variable** | **Overall** | **Returned to the same pre-admission functional category** | **Did not return to the pre-admission functional category** |
| --- | --- | --- | --- |
| Length of stay, days | 16.0 [11.0, 24.0] | 14.0 [11.0, 19.0] | 17.5 [11.0, 26.2] |
| PT sessions | 11.0 [7.0, 15.0] | 11.0 [7.0, 14.0] | 12.0 [8.0, 15.0] |
| OT sessions | 8.0 [5.0, 11.0] | 8.0 [5.0, 11.0] | 7.0 [5.0, 11.0] |
| Total therapy sessions | 19.0 [13.0, 25.0] | 19.0 [13.0, 25.0] | 19.0 [14.0, 25.0] |
| **In-hospital complication** |  |  |  |
| - No | 299 (84.5%) | 143 (97.9%) | 156 (75.0%) |
| - Yes | 55 (15.5%) | 3 (2.1%) | 52 (25.0%) |
| **Early mobilization within 48 h** |  |  |  |
| - No | 56 (15.8%) | 2 (1.4%) | 54 (26.0%) |
| - Yes | 298 (84.2%) | 144 (98.6%) | 154 (74.0%) |
| **Any improvement from admission to discharge** |  |  |  |
| - No | 277 (78.2%) | 89 (61.0%) | 188 (90.4%) |
| - Yes | 77 (21.8%) | 57 (39.0%) | 20 (9.6%) |

Values are presented as n (%) for categorical variables and median [interquartile range] for continuous variables. Hospital-course variables are shown descriptively according to whether patients returned to the same pre-admission functional category and were not included in the primary admission-time association model because they may reflect post-admission clinical course. Any improvement from admission to discharge was defined as a discharge functional category better than the admission category on the five-level ordinal scale (numerically lower ordinal score).

**Table S4. Modified Poisson sensitivity models using alternative admission functional-severity measures**

| **Predictor** | **FIM model aRR (95% CI)** | **Barthel model aRR (95% CI)** |
| --- | --- | --- |
| Male sex, versus female | 0.82 (0.64–1.06) | 0.82 (0.64–1.06) |
| Infection, versus cardiovascular | 1.07 (0.70–1.63) | 1.21 (0.78–1.87) |
| Musculoskeletal/trauma, versus cardiovascular | 1.23 (0.91–1.65) | 1.20 (0.89–1.62) |
| Neurologic/other, versus cardiovascular | 1.47 (0.89–2.46) | 1.51 (0.90–2.52) |
| Ambulatory with assistance, versus ambulatory | 2.14 (1.49–3.07) | 2.18 (1.52–3.13) |
| Chairbound/bedbound, versus ambulatory | 5.42 (3.09–9.49) | 5.53 (3.27–9.35) |
| Age, per 10 years | 1.04 (0.93–1.15) | 1.03 (0.93–1.14) |
| BMI, per 5 kg/m² | 1.07 (0.91–1.27) | 1.06 (0.91–1.25) |
| Comorbidity count, per 1 | 1.02 (0.96–1.07) | 1.01 (0.96–1.07) |
| Documented cognitive impairment at admission | 0.90 (0.67–1.21) | 0.96 (0.71–1.30) |
| Admission FIM, per 10-point increase | 1.15 (1.06–1.24) | — |
| Admission Barthel Index, per 10-point increase | — | 1.20 (1.10–1.31) |

*aRRs and 95% confidence intervals were estimated using modified Poisson regression with robust variance. Each model included the same covariates as the primary model, except that ordinal admission functional severity was replaced by FIM admission score or Barthel Index admission score.*

**Table S5. Correlations among admission functional-severity measures**

| **Variable** | **admission_function_ord** | **fim_admission** | **barthel_admission** |
| --- | --- | --- | --- |
| admission_function_ord | 1 | -0.977 | -0.986 |
| fim_admission | -0.977 | 1 | 0.987 |
| barthel_admission | -0.986 | 0.987 | 1 |

*Pearson correlation coefficients are shown. These measures were highly correlated and sensitivity analyses using FIM admission or Barthel admission should therefore be interpreted as alternative parameterizations of admission severity rather than independent validation.*

**Table S6. Logistic-regression model-form sensitivity analysis**

| **Predictor** | **Adjusted OR (95% CI)** |
| --- | --- |
| Male sex, versus female | 0.66 (0.40–1.10) |
| Infection, versus cardiovascular | 1.56 (0.59–4.14) |
| Musculoskeletal/trauma, versus cardiovascular | 1.89 (1.00–3.58) |
| Neurologic/other, versus cardiovascular | 3.12 (0.86–11.35) |
| Ambulatory with assistance, versus ambulatory | 5.49 (2.55–11.86) |
| Chairbound/bedbound, versus ambulatory | 45.97 (11.57–182.69) |
| Age, per 10 years | 1.05 (0.86–1.29) |
| BMI, per 5 kg/m² | 1.14 (0.80–1.62) |
| Comorbidity count, per 1 | 1.05 (0.94–1.19) |
| Documented cognitive impairment at admission | 0.96 (0.50–1.84) |
| Admission functional severity, per one-level worse | 0.36 (0.23–0.58) |

*The logistic model used the same predictors as the primary modified Poisson model. It was included only as a model-form sensitivity analysis; odds ratios should not be interpreted as directly comparable with the primary adjusted risk ratios.*

**Table S7. Full regression model output**

*All p values are two-sided robust Wald p values. No multiplicity adjustment was applied because analyses were exploratory. β denotes the log risk ratio for modified Poisson models and the log odds ratio for the logistic model. Robust standard errors use HC0 covariance estimation.*

**Panel A. Primary modified-Poisson model**

*Modified Poisson regression with robust (HC0) variance.*

| **Predictor** | **β** | **Robust SE** | **aRR** | **95% CI** | **P value** |
| --- | --- | --- | --- | --- | --- |
| Intercept | -0.921 | 0.670 | 0.398 | 0.107–1.479 | 0.169 |
| Male sex, versus female | -0.196 | 0.127 | 0.822 | 0.640–1.056 | 0.125 |
| Infection, versus cardiovascular | 0.304 | 0.224 | 1.355 | 0.874–2.101 | 0.174 |
| Musculoskeletal/trauma, versus cardiovascular | 0.224 | 0.150 | 1.251 | 0.933–1.677 | 0.135 |
| Neurologic/other, versus cardiovascular | 0.471 | 0.257 | 1.602 | 0.969–2.649 | 0.066 |
| Ambulatory with assistance, versus ambulatory | 0.849 | 0.188 | 2.337 | 1.618–3.375 | 6.03e-06 |
| Chairbound/bedbound, versus ambulatory | 1.833 | 0.287 | 6.251 | 3.562–10.967 | 1.67e-10 |
| Age, per 10 years | 0.031 | 0.052 | 1.031 | 0.931–1.142 | 0.552 |
| BMI, per 5 kg/m² | 0.060 | 0.078 | 1.062 | 0.911–1.238 | 0.444 |
| Comorbidity count, per 1 | 0.014 | 0.027 | 1.014 | 0.961–1.070 | 0.606 |
| Documented cognitive impairment at admission | -0.024 | 0.151 | 0.976 | 0.726–1.312 | 0.871 |
| Admission functional severity, per one-level worse | -0.525 | 0.118 | 0.591 | 0.469–0.745 | 8.61e-06 |

Model information: Patients / outcome events: 354 / 146; Parameters / residual df: 12 / 342; Log likelihood: -252.092; AIC: 528.184; BIC (log likelihood): 574.616; Deviance: 212.184; Pearson chi-square: 212.570; McFadden pseudo-R²: 0.084.

**Panel B. Exploratory ambulatory-baseline subgroup model**

*Modified Poisson regression with robust (HC0) variance; outcome: discharge below baseline.*

| **Predictor** | **β** | **Robust SE** | **aRR** | **95% CI** | **P value** |
| --- | --- | --- | --- | --- | --- |
| Intercept | -0.041 | 0.535 | 0.959 | 0.336–2.740 | 0.938 |
| Male sex, versus female | 0.110 | 0.099 | 1.116 | 0.918–1.356 | 0.270 |
| Infection, versus cardiovascular | -0.346 | 0.212 | 0.708 | 0.467–1.072 | 0.103 |
| Musculoskeletal/trauma, versus cardiovascular | -0.246 | 0.116 | 0.782 | 0.623–0.981 | 0.033 |
| Neurologic/other, versus cardiovascular | -0.233 | 0.227 | 0.792 | 0.507–1.236 | 0.304 |
| Age, per 10 years | -0.014 | 0.036 | 0.986 | 0.919–1.057 | 0.694 |
| BMI, per 5 kg/m² | -0.045 | 0.067 | 0.956 | 0.838–1.090 | 0.501 |
| Comorbidity count, per 1 | -0.034 | 0.026 | 0.966 | 0.918–1.017 | 0.192 |
| Documented cognitive impairment at admission | 0.009 | 0.133 | 1.009 | 0.777–1.311 | 0.945 |
| Admission functional severity, per one-level worse | 0.154 | 0.063 | 1.166 | 1.031–1.320 | 0.015 |

Model information: Patients / outcome events: 164 / 118; Parameters / residual df: 10 / 154; Log likelihood: -154.996; AIC: 329.991; BIC (log likelihood): 360.990; Deviance: 73.991; Pearson chi-square: 45.783; McFadden pseudo-R²: 0.012.

**Panel C. FIM admission sensitivity model**

*Modified Poisson regression with robust (HC0) variance.*

| **Predictor** | **β** | **Robust SE** | **aRR** | **95% CI** | **P value** |
| --- | --- | --- | --- | --- | --- |
| Intercept | -3.468 | 0.814 | 0.031 | 0.006–0.154 | 2.06e-05 |
| Male sex, versus female | -0.197 | 0.129 | 0.821 | 0.638–1.057 | 0.127 |
| Infection, versus cardiovascular | 0.069 | 0.215 | 1.071 | 0.702–1.633 | 0.750 |
| Musculoskeletal/trauma, versus cardiovascular | 0.204 | 0.152 | 1.227 | 0.911–1.653 | 0.179 |
| Neurologic/other, versus cardiovascular | 0.389 | 0.260 | 1.475 | 0.886–2.456 | 0.135 |
| Ambulatory with assistance, versus ambulatory | 0.760 | 0.185 | 2.138 | 1.487–3.074 | 4.09e-05 |
| Chairbound/bedbound, versus ambulatory | 1.690 | 0.286 | 5.419 | 3.093–9.493 | 3.47e-09 |
| Age, per 10 years | 0.036 | 0.054 | 1.036 | 0.933–1.151 | 0.508 |
| BMI, per 5 kg/m² | 0.071 | 0.084 | 1.074 | 0.911–1.265 | 0.396 |
| Comorbidity count, per 1 | 0.016 | 0.029 | 1.016 | 0.961–1.074 | 0.578 |
| Documented cognitive impairment at admission | -0.107 | 0.151 | 0.899 | 0.669–1.208 | 0.479 |
| FIM admission score, per 10 points | 0.137 | 0.038 | 1.147 | 1.064–1.237 | 3.30e-04 |

Model information: Patients / outcome events: 354 / 146; Parameters / residual df: 12 / 342; Log likelihood: -255.533; AIC: 535.066; BIC (log likelihood): 581.497; Deviance: 219.066; Pearson chi-square: 208.088; McFadden pseudo-R²: 0.072.

**Panel D. Barthel Index admission sensitivity model**

*Modified Poisson regression with robust (HC0) variance.*

| **Predictor** | **β** | **Robust SE** | **aRR** | **95% CI** | **P value** |
| --- | --- | --- | --- | --- | --- |
| Intercept | -3.442 | 0.776 | 0.032 | 0.007–0.147 | 9.24e-06 |
| Male sex, versus female | -0.195 | 0.129 | 0.823 | 0.639–1.059 | 0.130 |
| Infection, versus cardiovascular | 0.190 | 0.222 | 1.210 | 0.783–1.870 | 0.392 |
| Musculoskeletal/trauma, versus cardiovascular | 0.180 | 0.153 | 1.197 | 0.887–1.617 | 0.240 |
| Neurologic/other, versus cardiovascular | 0.411 | 0.261 | 1.508 | 0.904–2.517 | 0.116 |
| Ambulatory with assistance, versus ambulatory | 0.781 | 0.183 | 2.183 | 1.524–3.126 | 2.03e-05 |
| Chairbound/bedbound, versus ambulatory | 1.710 | 0.268 | 5.528 | 3.269–9.349 | 1.78e-10 |
| Age, per 10 years | 0.029 | 0.053 | 1.030 | 0.929–1.142 | 0.576 |
| BMI, per 5 kg/m² | 0.061 | 0.081 | 1.063 | 0.906–1.246 | 0.455 |
| Comorbidity count, per 1 | 0.013 | 0.028 | 1.013 | 0.959–1.070 | 0.644 |
| Documented cognitive impairment at admission | -0.040 | 0.153 | 0.961 | 0.712–1.297 | 0.794 |
| Barthel Index admission score, per 10 points | 0.184 | 0.046 | 1.202 | 1.099–1.315 | 5.69e-05 |

Model information: Patients / outcome events: 354 / 146; Parameters / residual df: 12 / 342; Log likelihood: -254.212; AIC: 532.424; BIC (log likelihood): 578.856; Deviance: 216.424; Pearson chi-square: 207.712; McFadden pseudo-R²: 0.077.

**Panel E. Logistic model-form sensitivity analysis**

*Logistic regression with robust (HC0) variance; odds ratios are not directly comparable with primary risk ratios.*

| **Predictor** | **β** | **Robust SE** | **OR** | **95% CI** | **P value** |
| --- | --- | --- | --- | --- | --- |
| Intercept | -0.516 | 1.412 | 0.597 | 0.038–9.496 | 0.715 |
| Male sex, versus female | -0.418 | 0.260 | 0.659 | 0.395–1.097 | 0.108 |
| Infection, versus cardiovascular | 0.445 | 0.498 | 1.561 | 0.589–4.138 | 0.371 |
| Musculoskeletal/trauma, versus cardiovascular | 0.636 | 0.326 | 1.888 | 0.996–3.578 | 0.051 |
| Neurologic/other, versus cardiovascular | 1.138 | 0.659 | 3.120 | 0.857–11.354 | 0.084 |
| Ambulatory with assistance, versus ambulatory | 1.704 | 0.392 | 5.494 | 2.546–11.858 | 1.42e-05 |
| Chairbound/bedbound, versus ambulatory | 3.828 | 0.704 | 45.967 | 11.566–182.691 | 5.41e-08 |
| Age, per 10 years | 0.052 | 0.103 | 1.053 | 0.860–1.289 | 0.618 |
| BMI, per 5 kg/m² | 0.131 | 0.178 | 1.139 | 0.804–1.615 | 0.463 |
| Comorbidity count, per 1 | 0.053 | 0.061 | 1.055 | 0.935–1.190 | 0.385 |
| Documented cognitive impairment at admission | -0.039 | 0.331 | 0.962 | 0.503–1.841 | 0.907 |
| Admission functional severity, per one-level worse | -1.011 | 0.241 | 0.364 | 0.227–0.583 | 2.61e-05 |

Model information: Patients / outcome events: 354 / 146; Parameters / residual df: 12 / 342; Log likelihood: -196.516; AIC: 417.032; BIC (log likelihood): 463.464; Deviance: 393.032; Pearson chi-square: 401.929; McFadden pseudo-R²: 0.181.

**Panel F. Exploratory split-category sensitivity model**

*Modified Poisson regression with robust (HC0) variance; chairbound and bedbound were separated for exploratory sensitivity analysis.*

| **Predictor** | **β** | **Robust SE** | **aRR** | **95% CI** | **P value** |
| --- | --- | --- | --- | --- | --- |
| Intercept | -0.986 | 0.663 | 0.373 | 0.102–1.367 | 0.137 |
| Male sex, versus female | -0.174 | 0.122 | 0.841 | 0.662–1.068 | 0.155 |
| Infection, versus cardiovascular | -0.032 | 0.256 | 0.969 | 0.586–1.601 | 0.902 |
| Musculoskeletal/trauma, versus cardiovascular | 0.300 | 0.152 | 1.350 | 1.002–1.817 | 0.048 |
| Neurologic/other, versus cardiovascular | 0.476 | 0.257 | 1.609 | 0.972–2.662 | 0.064 |
| Ambulatory with assistance, versus ambulatory | 0.862 | 0.186 | 2.367 | 1.645–3.406 | 3.44e-06 |
| Chairbound, versus ambulatory | 1.526 | 0.296 | 4.599 | 2.576–8.211 | 2.48e-07 |
| Bedbound, versus ambulatory | 2.386 | 0.350 | 10.874 | 5.475–21.598 | 9.37e-12 |
| Age, per 10 years | 0.024 | 0.051 | 1.024 | 0.926–1.132 | 0.644 |
| BMI, per 5 kg/m² | 0.064 | 0.078 | 1.066 | 0.915–1.243 | 0.411 |
| Comorbidity count, per 1 | 0.022 | 0.026 | 1.022 | 0.971–1.076 | 0.404 |
| Documented cognitive impairment at admission | 0.027 | 0.140 | 1.028 | 0.780–1.353 | 0.846 |
| Admission functional severity, per one-level worse | -0.520 | 0.119 | 0.595 | 0.471–0.751 | 1.26e-05 |

Model information: Patients / outcome events: 354 / 146; Parameters / residual df: 13 / 341; Log likelihood: -248.689; AIC: 523.379; BIC (log likelihood): 573.680; Deviance: 205.379; Pearson chi-square: 219.494; McFadden pseudo-R²: 0.097.

**Table S8. Primary modified-Poisson model diagnostics and robustness checks**

*Diagnostics were calculated from the fully adjusted primary modified-Poisson model including sex and exact age. Summary-level diagnostic results are reported here; individual-level restricted data are not included in the public Supporting Information.*

**Panel A. Model convergence, fit, and fitted values**

| **Diagnostic** | **Result** |
| --- | --- |
| Patients / outcome events | 354 / 146 |
| IRLS convergence | Converged after 5 iterations |
| Residual deviance / residual degrees of freedom | 212.184 / 342 (0.620) |
| Pearson chi-square / residual degrees of freedom | 212.570 / 342 (0.622) |
| Fitted-value range | 0.051 to 1.449 |
| Fitted values >1 | 11 of 354 (3.1%) |
| Maximum leverage | 0.269 |
| Maximum Cook's distance | 0.0220 |

**Panel B. Collinearity assessment**

| **Predictor** | **Variance inflation factor** |
| --- | --- |
| Infection, versus cardiovascular | 3.54 |
| Musculoskeletal/trauma, versus cardiovascular | 1.54 |
| Neurologic/other, versus cardiovascular | 1.20 |
| Ambulatory with assistance, versus ambulatory | 1.51 |
| Chairbound/bedbound, versus ambulatory | 2.82 |
| Male sex, versus female | 1.09 |
| Age, per 10 years | 1.73 |
| BMI, per 5 kg/m² | 1.20 |
| Comorbidity count, per 1 | 1.86 |
| Documented cognitive impairment at admission | 1.40 |
| Admission functional severity | 3.49 |

**Panel C. Functional-form checks using quadratic terms**

| **Quadratic term** | **β** | **Robust SE** | **aRR** | **95% CI** | **P value** |
| --- | --- | --- | --- | --- | --- |
| Age, per 10 years squared | -0.005 | 0.020 | 0.995 | 0.958-1.035 | 0.811 |
| BMI, per 5 kg/m² squared | -0.014 | 0.083 | 0.986 | 0.838-1.161 | 0.870 |
| Comorbidity count squared | -0.002 | 0.005 | 0.998 | 0.989-1.008 | 0.749 |

**Table S8. Continued**

**Panel D. Influence sensitivity analysis**

*Observations with Cook's distance >4/n (threshold 0.0113) were excluded (5 observations; 349 patients and 142 outcome events remained).*

| **Key association** | **Full model aRR (95% CI)** | **Influence-excluded model aRR (95% CI)** |
| --- | --- | --- |
| Ambulatory with assistance, versus ambulatory | 2.337 (1.618-3.375) | 2.544 (1.748-3.704) |
| Chairbound/bedbound, versus ambulatory | 6.251 (3.562-10.967) | 8.684 (4.895-15.407) |
| Admission functional severity, per one-level worse | 0.591 (0.469-0.745) | 0.499 (0.395-0.631) |

*aRR, adjusted risk ratio; IRLS, iteratively reweighted least squares. Fitted values above 1 can occur in modified-Poisson models and are reported transparently here; robust variance was used for all modified-Poisson estimates.*
